# Oxidative Stress and Photodynamic Therapy of Skin Cancers: Mechanisms, Challenges and Promising Developments

**DOI:** 10.3390/antiox9050448

**Published:** 2020-05-22

**Authors:** Alessandro Allegra, Giovanni Pioggia, Alessandro Tonacci, Caterina Musolino, Sebastiano Gangemi

**Affiliations:** 1 Division of Haematology, Department of Human Pathology in Adulthood and Childhood “Gaetano Barresi”, University of Messina, 98125 Messina, Italy; cmusolino@unime.it; 2 Institute for Biomedical Research and Innovation (IRIB), National Research Council of Italy (CNR), 98164 Messina, Italy; giovanni.pioggia@cnr.it; 3 Clinical Physiology Institute, National Research Council of Italy (IFC-CNR), 56124 Pisa, Italy; atonacci@ifc.cnr.it; 4 School and Operative Unit of Allergy and Clinical Immunology, Department of Clinical and Experimental Medicine, University of Messina, 98125 Messina, Italy; gangemis@unime.it

**Keywords:** oxidative stress, photodynamic therapy, skin cancer, reactive oxygen species, carcinogenesis

## Abstract

Ultraviolet radiation is one of the most pervasive environmental interactions with humans. Chronic ultraviolet irradiation increases the danger of skin carcinogenesis. Probably, oxidative stress is the most important mechanism by which ultraviolet radiation implements its damaging effects on normal cells. However, notwithstanding the data referring to the negative effects exerted by light radiation and oxidative stress on carcinogenesis, both factors are used in the treatment of skin cancer. Photodynamic therapy (PDT) consists of the administration of a photosensitiser, which undergoes excitation after suitable irradiation emitted from a light source and generates reactive oxygen species. Oxidative stress causes a condition in which cellular components, including DNA, proteins, and lipids, are oxidised and injured. Antitumor effects result from the combination of direct tumour cell photodamage, the destruction of tumour vasculature and the activation of an immune response. In this review, we report the data present in literature dealing with the main signalling molecular pathways modified by oxidative stress after photodynamic therapy to target skin cancer cells. Moreover, we describe the progress made in the design of anti-skin cancer photosensitisers, and the new possibilities of increasing the efficacy of PDT via the use of molecules capable of developing a synergistic antineoplastic action.

## 1. Introduction

### Ultraviolet Radiation, Oxidative Stress and Skin Cancer

**Figure 1:**
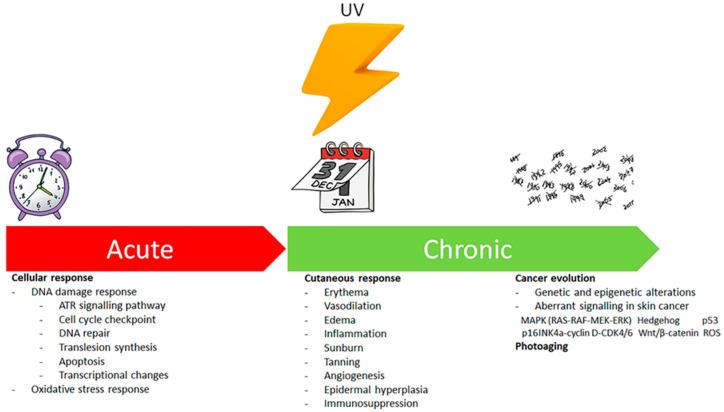
Acute and chronic effects of ultraviolet (UV) radiation.

Ultraviolet radiation (UVR) is one of the most pervasive environmental interactions with humans. UV exposure mainly influences the skin in humans and is able to induce the appearance of skin erythema and vasodilation, inflammation, immunosuppression, dermatoheliosis and epidermal hyperplasia [1,2,3,4,5,6,7,8,9] [Table 1]. Moreover, chronic Ultraviolet (UV) irradiation increases the danger of skin carcinogenesis [10] (Figure 1).

Although exposure to UVR produces harmful effects, UVR should be separated into four ranges—UVA1 (340–400 nm), UVA2 (320–340 nm), UVB (280–320 nm) and UVC (200–280 nm)—with the majority of UVR reaching the skin’s surface falling into the former three categories due to the filtering effects of atmospheric ozone. For this reason, the light of each of these regions results in rather different tissue effects, not all of which are associated with carcinogenesis. Because minimal UVC reaches the Earth’s surface, the majority of direct DNA damage is attributed to radiation in the UVB spectrum. DNA has an absorption peak in the UVC region at approximately 260 nm, with significant absorption in the UVB and in the UVA regions as well. [11].

Exposure to UVR damages epidermal DNA via several mechanisms [12]. The absorption of energy in the UVB spectrum causes characteristic photoproducts, the most common of which are the cyclobutane pyrimidine dimers (CPDs). CPDs are formed between the C-4 and C-5 carbon atoms of two adjacent pyrimidines, with double bonds becoming saturated to give rise to a four-member ring. Subtypes of CPDs include thymine-thymine (T=T), cytosine-cytosine (C=C), thymine-cytosine (T=C) and cytosine-thymine (C=T). When nucleotide excision enzymes fail to repair these modifications and DNA polymerases attempt to replicate the structurally altered DNA, the polymerases insert adenines opposite these bulky photoproducts [13]. In the case of T=T dimers, there are no resulting mutations, as A is normally paired with T. However, in the case of C=C CPDs, a CC > TT transition occurs, resulting in a mutated DNA sequence. Thus, areas of the genome with a high frequency of adjacent pyrimidines are considered “UV hotspots” and show high rates of C > T and CC > TT “UV signature” mutations [14]. Indirect damage, by contrast, begins when a photophore in the epidermis other than DNA is excited by UVR. Such endogenous molecules include tryptophan, riboflavin, porphyrins and melanin, among others. When these molecules operate a transition from a UVR-excited state back to resting state, energy may be transferred to DNA, causing structural changes indistinguishable from those of direct DNA damage [15].

There are several systems by which UV radiation implements its damaging effects on normal cells. Probably, oxidative stress is the most important of these mechanisms. Reactive oxygen species (ROS) are unstable molecules that include oxygen and they promptly react with several other molecules in cells. ROS are produced in cells because of cellular metabolism, but they are also produced by external elements including UV irradiation [16]. Remarkably, visible light (400–700 nm wavelength) also generates ROS [17]. These molecules include hydrogen peroxide, superoxide and the hydroxyl radical. UV radiation causes extreme quantities of ROS that overcome antioxidant systems like those represented by glutathione. In skin cells and, above all, in melanocytes, the melanosome is probably the main source of ROS [18]. Moreover, the extracellular matrix (ECM) of the skin is probably an important source of ROS after UV exposure [19]. When ECM proteins, such as elastin and collagen, were added to dermal fibroblasts after pre-irradiation with UV, dermal fibroblasts displayed an increase in oxidative stress [19]. This demonstrates that ECM proteins operate like photosensitisers to produce ROS after UV irradiation.

The presence of unpaired electrons in free radicals provides high reactivity, which induces the beginning of reduction–oxidation (redox) reactions with the potential to damage crucial biomolecules. Cellular redox homeostasis is generally preserved by a subtle delicate equilibrium between ROS production and antioxidant defense systems. When this balance is altered in biological systems, oxidative stress occurs from the overproduction of ROS. Cellular injury resulting from excessive ROS generation represents a consequence of interference with three major cellular components: cellular membranes, proteins and DNA. In this regard, oxidative stress damages cellular components with the consequent alteration of overall biological activities [20]. Both the superoxide anion and hydroxyl radical species can cause the oxidative degradation of cellular membranes in a process known as lipid peroxidation. The reaction of these radicals with polyunsaturated fatty acids (lipids) in cellular membranes causes peroxyl radicals, which lead to the downstream production of additional free radicals and over 200 cytotoxic reactive aldehyde species [21]. The second main target of ROS is represented by proteins, specifically the amino acid residues cysteine and methionine, which are prone to oxidation by ROS. The oxidation of protein sites critical to structural or enzymatic activity finally results in protein inactivation or misfolding [22]. Finally, DNA is also a major target of ROS, as we reported above.

Excessive concentrations of ROS promote an altered cell growth, DNA damage and epigenetic modifications, and cause the onset of several diseases including tumours [23,24]. An oncogenic action of ROS is confirmed in Xeroderma pigmentosum group C (XPC) deficiency. XPC loss provokes ROS-caused alterations in cellular metabolism and modifications of several signalling pathways that are linked to carcinogenesis. For instance, ROS are able to modify several pathways that are activated in several tumours including the activating protein-1 pathway, epidermal growth factor receptor, NF-κB, mitogen-activated protein kinase/extracellular signal-regulated kinase (MAPK/ERK), and p38 MAPK [16,25,26,27]. The stimulation of NF-E2-related factor 2 (NRF2), a transcription element that activates antioxidant systems, reduces ROS concentrations and prevents ROS-caused DNA alterations after UV exposure, thus blocking the process of skin carcinogenesis [28]. However, disproportionate NRF2 activation may be disadvantageous, as an excessive amount of active NRF2 causes corneocyte fragility and increased immune infiltration into the skin in transgenic mice [28] (Table 1).

As described in the previous paragraphs, the alteration of oxidative stress can be of paramount importance in the onset of skin diseases, both neoplastic and non-neoplastic [29,30,31,32,33,34,35,36]. It may therefore come as a surprise that the stimulation of oxidative stress can be used for the treatment of neoplastic pathologies and of skin ones.

Indeed, even though a mutagenic action of UV-enhanced ROS has been established, experimental data revealed that oxidative stress blocked melanoma metastasis in an animal model [37]. In this report, circulating melanoma cells and the melanoma cells present in visceral metastatic nodules were subjected to increased concentrations of ROS. Melanoma cells presented an enhanced level of nicotinamide adenine dinucleotide phosphate (NADPH)-generating enzymes that are implicated in antioxidant effects. The external addition of antioxidants increased the metastasis of melanoma cells, while the reduction of NADPH-producing enzymes reduced metastatic diffusion. This report proposes that, as opposed to ROS-caused oncogenic signalling, oxidative stress in melanoma cells blocks metastasis [38].

## 2. Photodynamic Therapy of Skin Cancer and Oxidative Stress

As for skin cancer, the main forms of skin tumour include basal cell carcinoma (BCC), cutaneous squamous cell carcinoma (cSCC), and melanoma. BCC and cSCC are also known as nonmelanoma skin cancer (NMSC). The annual occurrence of NMSC is 5.4 million in the United States [39].

Melanoma is an extremely aggressive skin carcinoma representing the main cause of death associated with skin tumours [40]. Even though targeted treatments and immunotherapy have shown to cause an increase in the overall survival and progression-free survival of melanoma subjects, long-lasting responses happen in few patients [41], and novel treatment options need to be investigated.

Notwithstanding the above reference to the negative effects exerted by light radiation and oxidative stress on carcinogenesis and tumour progression, both factors are used in the treatment of neoplastic diseases (Table 1).

The take-off of photodynamic therapy (PDT) goes back to the first years of the 20th century, when it was detected that microorganisms incubated with acridyne dyes died after light exposure [42]. Shortly after, PDT was employed to treat a skin cancer, applying eosin and visible light [43]. Hematoporphyrin-derived products separated from porcine blood represented the first photosensitiser (PS) accepted for human practice [44].

In the late 1970s, it was demonstrated that PDT could treat cancers in animal experimental models [45], and a little later, the effectiveness of PDT for human cancer was confirmed [46]. In the 1990s, PDT was authorised for the treatment of oesophageal cancer. Later, PDT with blue light and 5-aminolevulinic acid (5-ALA) was accepted by the FDA to treat actinic keratoses (AKs) of the face and scalp, while PDT with blue light was accepted for BCC and cSCC in situ in Europe. PDT has been also demonstrated to be efficient in the treatment of invasive cSCC [47,48,49,50]. Furthermore, PDT has been employed to treat tumours of the genitourinary and gastrointestinal tracts and for lung cancer [51], as well as for the treatment of head and neck tumours.

PDT is a negligibly intrusive treatment that merges the use of a non-toxic PS and visible light to provoke a specific cytotoxic action activity towards neoplastic cells [52,53,54].

**Figure 2:**
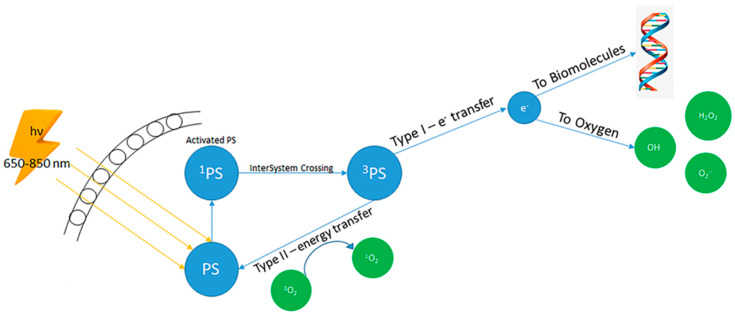
Mechanisms of photodynamic therapy. When a photosensitiser (PS) absorbs a photon, it is excited to the singlet state (^1^PS), then it will form a long-lived triplet state (^3^PS). A type I reaction drives the ^3^PS to transfer an electron to biomolecules or directly to oxygen, forming radicals able to react with oxygen. A type II reaction involves energy transfer from the excited PS to molecular oxygen.

Irradiation at a wavelength equivalent to an absorbance band of the PS causes its excitation, which produces ROS by two main actions, identified as type I and type II reactions [55,56]. A type I reaction happens when a PS interacts with an organic element to develop radicals, such as hydrogen peroxide, superoxide anion, and hydroxyl radicals. Differently, in type II reactions, a PS transmits its energy to molecular oxygen, provoking the creation of singlet oxygen, a molecule with an extremely high cytotoxic activity [57] (Figure 2). The increase in ROS causes oxidative stress, a situation in which cellular components, including DNA, proteins and lipids, are oxidised and injured [58]. The survival of the exposed organism is determined by the capacity of cells to oppose the stress and eliminate or fix injured elements. Several stress response systems are promptly actuated in response to oxidative offences, including the activation of enzymatic and non-enzymatic antioxidising factors [59,60]. However, ROS stimulate diverse intracellular signalling pathways and can cause several effects including proliferation block and cell death [61]. The type of effect is influenced by the cell type, the stimulus exerted, its intensity and its extent [62]. As mentioned above, oxidative stress can influence the activity of several pathways capable of affecting cell survival and proliferation. For instance, it was proposed that ROS might activate the MAPK and PI3K pathways through the oxidative modification of intracellular kinases [63,64,65,66,67,68]. The control exerted by oxidative stress at the molecular level justifies the actions it exerts on cancer cells.

### 2.1. PDT, Oxidative Stress and Cellular Death

Some research on PDT’s actions was centred on cell death, due to the production of ROS. However, several factors seem to be able to influence the effectiveness of PDT and its ability to induce cell death.

The superoxide ion is one of the main results of phototoxic reactions. Owing to the extremely short half-life, calculated in nanoseconds, this cytotoxic element can circulate only in a very restricted range, up to 20 nm in cells [69]. Thus, the subcellular position of the PS controls which organelles are mainly harmed, and intracellular targeting is a challenge seen in the impediment in attaining adequate diffusion into the cell.

The melanin amount can also modify the efficacy of PDT. Employing three different bacteriochlorins as PSs, it was demonstrated that all bacteriochlorins are stored in melanosomes, provoking melanosomal damage. The less efficacious bacteriochlorins are essentially located in lysosomes, while the more efficacious ones are mainly stored in mitochondria and generate drastically greater concentrations of hydroxyl radicals [70]. The melanosomal storage of the PSs in the melanoma cells might cause the damage of such organelles, leading to cell death by several factors including H_2_O_2_ and highly active quinones [71].

A different element capable of influencing the effectiveness of PDT is the presence of antioxidant systems. Superoxide dismutase (SOD), as both the constitutive (Cu, Zn-SOD) and an inducible (MnSOD) isoform, is involved in superoxide ion scavenging. PDT increases the production of the Mn-SOD but not of the Cu, Zn-SOD isoform [72]. Several studies propose a relevant protective action of the inducible Mn-SOD isoform of SOD in cancer cells exposed to PDT [73,74].

Finally, although PDT is used to destroy neoplastic cells, the intimate mechanisms of such events are not fully known [75]. After absorbing light, PSs generate ROS, causing programmed cell death, necrosis, autophagy, inflammation and a general immune reaction against cancer cells [54]. This ROS-provoked cancer cell killing mechanism is analogous to the effect of some chemotherapy drugs such as bleomycin [76,77].

As reported above, the cell death caused by PDT can happen either by programmed cell death or by necrosis, changing depending on the cell type; oxygen concentration; and the type, amount and intracellular storage of the PS, as well as the light dose and the wavelength of the light [78,79,80]. The production of great concentrations of oxidative stress causes the onset of necrotic cell death, while minor oxidative stress induces apoptosis. Moreover, sublethal doses of PDT may induce the alteration of cell surface receptors and modify cell functions.

Apoptosis is a regulated process of suicidal cell death due to the stimulation of hydrolytic enzymes such as nucleases and proteases, provoking DNA destruction and the alteration of intracellular structures. The apoptotic event may be stimulated by mitochondrial-dependent systems or by the receptors in the cell membrane employing pathways implicating c-AMP, calcium ions, protein kinases, ceramides and transcription factors. Caspase-3 is the main effector, being responsible for the activation of the other caspases and some nuclear components implicated in DNA repair. Apoptosis has been demonstrated to be the main form of cell death following PDT in different experimental situations employing different PSs and cell types [81,82,83].

PDT causes programmed cell death in two ways: the intrinsic pathway and extrinsic pathway [84]. The mitochondrial apoptosis pathway is mainly triggered when PSs are stored inside the mitochondria, with the subsequent appearance of the disturbance of the mitochondrial transmembrane potential and the delivery of cytochrome C into the cytosol. These alterations cause the creation of a system named the apoptosome and provoke the stimulation of caspases 2, 3, 6, 7 and 8, demonstrated to be, in turn, stimulated after PDT [85,86,87]. Death receptor-provoked apoptosis happens especially when PSs affect the cell membrane. It is caused by the multimerisation of cell membrane receptors belonging to the TNF receptor (TNFR) superfamily, particularly Fas receptor. The death provoking signalling complex (DISC) established by Fas receptor, Fas-associated death domain protein and caspase 8 is a critical element of programmed cell death [88]. The action of p53 in PDT in causing apoptosis in cancer cells is not completely understood; indeed, even though the PDT of cancer cells provokes an increased production of p53, the cell death might be p53-independent [89].

However, although apoptosis is the form of cell death examined in a greater number of investigations, it is possible that some cells may also experience necrosis after PDT.

From this point of view, the cell type implicated is critical to the nature of the cell death that is provoked. For instance, PDT employing hypericin stimulated by ultraviolet A (UVA) irradiation caused a necrotic cell death in pigmented melanoma cells and a programmed cell death in keratinocytes and non-pigmented melanoma cells [90].

Factors that promote necrosis include the extra-mitochondrial localisation of the PS, a high dose of PDT, glucose starvation and the cell genotype [91]. In the case of necrosis, cytosolic constituents spill into the extracellular space through the damaged plasma membrane and provoke a robust inflammatory response. As we will be better discussed later, this inflammation might potentiate immunity by attracting host leukocytes into the tumour and increasing antigen presentation. Moreover, necrosis leads to a more important immunological activation, and this could be beneficial for the final outcome of PDT, especially in melanoma, where the presence of a higher frequency of tumour-infiltrating lymphocytes is associated with a better outcome [92,93].

**Figure 3:**
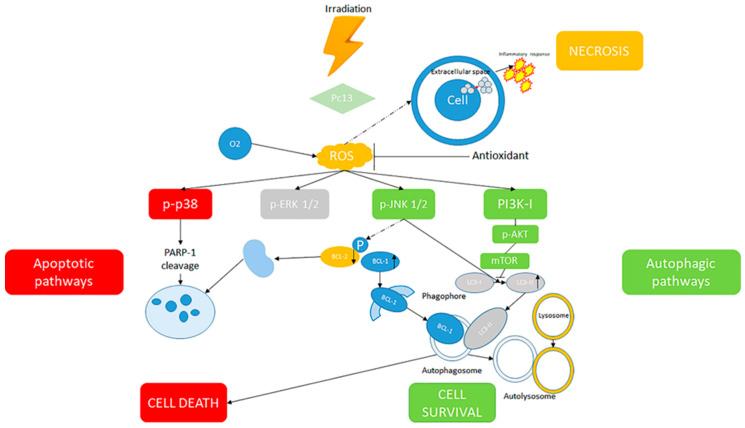
Mechanisms of cell death, survival and necrosis from irradiation.

The increase in ROS can also stimulate a third type of cell death, autophagy, an event involving a selective destruction of cellular elements (Figure 3). Under stress situations, autophagy has a pro-survival action implicated in the renewal of proteins and removal of injured organelles to preserve cell health [94]. However, disproportionate autophagy or the incongruous stimulation of autophagy may cause cell death [95]. In any case, the generation of an autophagic response has been revealed after PDT, but a double action has been reported, since it may cause either a survival stimulus or an increased death pathway [96].

### 2.2. Molecular Mechanisms of Cell Death

Some studies have attempted to justify the different types of cell death at the molecular level. The blockade of p38 decreased Pc13 phototoxicity, while the inhibition of ERK did not alter this response. On the other hand, JNK blockade increased the action of Pc13 PDT. Data obtained demonstrate that p38 is implicated in the cleavage of PARP-1, a central effector of apoptosis. Conversely, Pc13 irradiation caused the stimulation of an autophagic programme, as demonstrated by the increased concentrations of LC3, GFP-LC3 and Beclin-1 punctate staining. It was also reported that this autophagic response is increased by JNK and reduced by the PI3K-I/AKT pathway. The inhibition of autophagy enhanced Pc13 phototoxicity and increased PARP-1 cleavage, showing a protective action of this mechanism, which tries to inhibit apoptotic cell death. Moreover, a decreased vulnerability to treatment and the augmented stimulation of autophagy were demonstrated in A375 cells subjected to reiterated cycles of Pc13 PDT, suggesting that autophagy could be a means of resistance to PDT.

In conclusion, the relationship between programmed cell death and the autophagic response stimulated by Pc13 PDT-provoked oxidative stress was confirmed. Consequently, autophagy control might be a favourable therapeutic option to increase the effectiveness of PDT in melanoma patients [97].

### 2.3. Additional Mechanisms of Action of PDT

In addition to the cytotoxic effects, PDT could exert its beneficial effects through other mechanisms. Different experiments conducted in vivo on diseases other than skin cancers have revealed that the positive action exerted by oxidative stress after PDT could be due to its action on the immune system.

Filip et al. studied the actions of PDT with 5,10,15,20-tetrakis(4-methoxyphenyl)-porphyrin (TMPP) and its zinc complex (ZnTMPP) on the cancer concentrations of cytokines, malondialdehyde (MDA) and reduced glutathione (GSH) and made an attempt to link them with the histological modifications of cancer tissues in Wistar rats carrying Walker 256 carcinosarcomas. The cancer tissue concentrations of TNF-α and MDA were considerably greater in treated cancers than in controls, and these results were related to the histological modifications. These data propose that PDT stimulates the innate immune system and that this action is due to ROS production [85].

### 2.4. PSs, Lipid Peroxidation and PDT

However, related to the sort of PS, the onset of a different type of oxidative stress can also be stimulated, capable of influencing the glucose, protein and lipid metabolism of cancer cells. For instance, there are several in vivo and in vitro reports demonstrating that PDT causes increased concentrations of lipid peroxides [98]. Moreover, it was reported in diverse cell lines that lipid peroxidation occurs within a few minutes after PDT [99].

The different types of oxidation, including that of the cellular lipid membrane, could also provoke diverse mechanisms of action of PDT with a modification of the ratio of the stimulation of cell death by apoptosis or by necrosis. MDA is a marker employed to study lipid peroxidation in tissues. Several experiments demonstrated that the MDA concentrations in cancer tissues were increased after ZnTMPP or TMPP PDT with respect to tissues in control groups. Moreover, TMPP PDT and ZnTMPP PDT led to an increased concentration of thiobarbituric reactive substances (TBARS), a parameter of lipid peroxidation, in tumour tissue and in blood plasma at 24 h after the PDT.

Moreover, glutathione concentrations in cancer increased significantly after ZnTMPP and TMPP PDT with respect to 5-ALA PDT. It is likely to be able to ascribe the augmentation of GSH concentrations to the increase in lipid peroxides in cells. In this regard, several studies have been conducted to evaluate the role of glutathione in PDT. In fact, it has been demonstrated that PDT, via NF-kB, provoked an increase in γ-glutamylcysteine synthetase production in response to oxidative stress-caused GSH depletion [100,101,102]. Furthermore, it has been demonstrated that the stimulation of apoptosis-performing caspases needs adequate glutathione concentrations as these proteases have a crucial cysteine thiol in the active site [100]. A direct correlation between the action of caspase-3 and glutathione concentrations after 5-ALA PDT has been revealed and supports this hypothesis. The SH groups of caspases are pivotal for their catalytic action. Due to exposure to free radicals, such SH groups might have been deactivated.

## 3. Novel Photosensitisers

Other PSs have been employed to improve PDT effects; below are some of the most promising experimental studies that have correlated the effectiveness of PDT with changes in oxidative stress in skin cancers, whose main findings are briefly mentioned in Table 1.

Research on PDT tries to create new PSs for enhanced tissue selectivity and light absorbance. To increase the effectiveness of PDT and amplify ROS generation, attempts are being made to increase light and drug diffusion into deeper tissue and the energy absorption by PSs [103]. Generally, PDT has been employed with one wavelength of either 400 nm (blue light) or 635 nm (red light) to excite a PS.

To establish the ideal wavelength for treating cancer, the features of diverse wavelengths must be studied. Compared to shorter wavelengths, longer ones might have higher diffusion into deeply invaded cancers but have less effectual light absorption by PSs. The commonly employed PS Protoporphyrin IX (PpIX), which is produced from a prodrug such as 5-ALA, has five absorption peaks at 410 nm, 510 nm, 545 nm, 580 nm and 630 nm, but only the last two peaks are correlated with penetrative light in skin. The concomitant use of 405 nm and 505 nm wavelengths produced more ROS than 405 nm alone in cells treated with 5-ALA [99]. In an animal experimental model, this dual-wavelength PDT inhibited cancer proliferation more efficiently than a 405 nm single-wavelength PDT [104].

For instance, metallo 5,10,15,20-tetrakis(4-methoxyphenyl) porphyrin (TMPP) is a tetrapyrrolic macrocycle with an elevated absorption coefficient in the visible spectrum and with a protracted life of the triplet state. Its structure, characterised by the presence of methoxy substituents, offers to this substance relevant photosensitising capacities, increasing its polarity but, at the same time, maintaining the lipophilic character due to the tetraphenyl-porphyrin, thus assuring an appropriate interaction with the tissue. The complexing with metals such as Zn (II) and Cd (II) increases the amount of singlet oxygen generated, causing an intensified photodynamic action [105,106].

Different information about the relationship between PDT and oxidative stress came from the studies conducted using as PSs novel indolines-fused-triazoles [100]. A research study analysed the signalling pathways implicated in the PDT action of triazoles on BCC cells under UVA irradiation [107]. The data demonstrated that the therapy of BCC cells with 1j-UVA caused an augmented ROS production, reduced concentrations of Bcl-2 and Bcl-xL, augmented concentrations of Bad and Bax, cytochrome c delivery and caspase-3/PARP degradation [107]. This evidence was validated by other authors. Hu et al. analysed the signalling pathways implicated in the PDT action of triazoles on BCC cells under UVA irradiation. Intracellular ROS and the mitochondrial membrane potential (ΔΨ_mt_) were evaluated, employing a DCFH-DA probe and DiOC_6_ dye. Their results revealed that the therapy of BCC cells with 1j-UVA provoked augmented ROS production and the loss of mmp (ΔΨ_mt_) [108].

Although this study has allowed to better define the pathways related to the apoptosis, other research has enabled the examination of the effects of oxidative stress on mitochondria, which are a probable target of PDT, as mentioned above [109,110]. For instance, Choromańska et al. evaluated the action of photodynamic reactions in human melanoma cell lines employing, as a PS, Photofrin (Ph) in vitro. The primary cell line used was the MEWO cell line, originating from a human melanoma. As a recurrent cell line, they employed the Me45 cell line, originating from a lymph node metastasis of skin melanoma. They found that Me45 and MEWO cell survival was related to the duration of the incubation after the exposure. In the recurrent cell line, the Ph was stored mainly in the mitochondrial membranes, while in MEWO cells, it was also in the cytoplasm. The primary melanoma cell line demonstrated a reduction of cellular growth (below 50%) after PDT with Ph. Moreover, they stated that the mitochondrial localisation of Ph is responsible for the alterations of the mitochondrial transmembrane potential and for the delivery of apoptotic proteins [111].

Finally, some studies aimed at detecting a different mechanism of action of oxidative stress during PDT appear particularly interesting. Clusterin, an extensively expressed glycoprotein, also called apolipoprotein J, has been demonstrated to be increased after chemical or cytotoxic injuries [112]. A quick increase in clusterin was highlighted at the onset of apoptosis in in vitro and in vivo experimental models [113]. Likewise, an increased production of clusterin was demonstrated after the administration of elevated quantities of TNF in a fibrosarcoma cell line with a constitutively small expression of clusterin [114]. The clusterin protein and transcript concentrations were also stimulated by small levels of ionising radiation in several rodent and human cancer cells [115]. Viard et al. have reported a relevant increase inclusterin mRNA concentrations in human epidermoid carcinoma cells A431 after oxidative stress due to the superoxide anion, hydrogen peroxide, hyperoxia, UVA radiation and transitory heat shock [116]. Furthermore, clusterin has been reported to be stimulated in tissues degenerating as an effect of oxidative stress-caused cell death. The management of programmed cell death-sensitive human epidermoid carcinoma cells (A431) with PDT provoked a relevant increase in clusterin with a peak at 12 h after treatment, while the clusterin concentrations in Pc 4-PDT-treated, apoptosis-resistant, radiation-induced fibrosarcoma (RIF-1) cells remained unaffected. The intravenous dispensation of Pc 4 and light application to animals carrying UVB radiation-caused skin papillomas provoked an augmentation of clusterin production, peaking at 24 h after the therapy, when the cancer reduction was evident [117]. Such results confirmed the participation of oxidative stress in clusterin PDT-mediated cell death and cancer regression.

## 4. Conclusions

It is evident from the above that PDT—through its main, though not exclusive, effect on oxidative stress—represents an effective option for the therapy of skin cancer. Compared to the traditional antitumor treatments such as chemotherapy, radiotherapy and surgery, PDT presents several advantages. It is essentially a non-invasive treatment, and it is site-specific, thus it can be employed when traditional treatment is unsuccessful or not advisable [118]. Moreover, the PSs employed are generally non-toxic in the absence of light and are passively stored in the cancer tissue [119].

Furthermore, recent research has made it possible to reduce the problems related to the technique. For instance, for much time, PDT has been considered to be a costly treatment due to the elevated costs of PS and light sources. At present, this is not a relevant difficulty as light sources such as light-emitting diodes (LEDs) are accessible at low cost. Moreover, the creation of a portable low irradiance organic LED improved the ambulatory therapy of skin tumours, and it is now possible to perform PDT in an out-patient setting that decreases the costs of patient treatment [120,121]. Furthermore, PDT is also effective in the therapy of chemo- and radio-resistant cancers, and it has not been described as a mutagenic treatment as none of the PSs underwent storage in the cell nucleus [122].

However, not all problems related to the use of phototherapy have been solved. The side effects of PDT include itching, exudation, oedema, erythema and aching after exposure to light. Several measures can be undertaken to reduce the side effects of PDT. For instance, local anaesthetic nerve blockers for pain reduction may be employed during PDT when the inconvenience is insupportable [123,124]. Nevertheless, novel approaches will have to be realised to decrease such negative effects of the treatment, which reduce the efficacy of and the possibility of employing phototherapy.

Furthermore, although PDT has been defined above as a non-invasive treatment, there are conditions in which the impact of this treatment is greater, for example, in the so-called interstitial PDT (I-PDT). This technique is a promising alternative treatment for patients with deep-seated or locally advanced cancers (≥10 mm in thickness). In I-PDT, the tissue-localised PS is activated with laser light delivered through multiple diffusing optical fibres, which can be inserted directly into the target tumour volume through sterilised transparent catheters. Specific problems related to this treatment will have to be addressed and resolved [125].

One aspect that needs to be explored soon is the possibility that some forms of PDT might exert a negative action on the immune system and on immunosurveillance.

For instance, excitement for the periodic broad-area short incubation (BASI)-PDT of subjects at elevated risk for new skin cancers has been reduced by the fact that PDT decreases immunocompetence in animals for at least 28 days [126]. In humans, in vivo studies have demonstrated that the MAL-PDT treatment decreases local recall immunity reactions for at least 24 h [127]. An analogous state of immunosuppression has been reported after UV exposure in mice and might be a relevant permissive element for human photocarcinogenesis. In both human and murine skin, this condition is due to a reduction in cutaneous Langerhans cells (LCs), which are known to act as the antigen-presenting cells accountable for the identification of tumour-associated neoantigens. The LC decrease after PDT is ascribed to the oxidative DNA damage provoked by the ROS produced during the treatment [128,129,130]. A dose–rate dependence of LC reduction and the immunosuppressive state have been reported for ALA-PDT. It is in fact noteworthy that, employing the standard irradiance of 75 mW/cm^2^, such alterations in the skin of human volunteers were produced, but lower irradiances of 15 or 45 mW/cm^2^ had no effect [128]. However, in another study, Ramaswamy et al. reported that PDT had no effects on the epidermal LC number or dentricity [131].

The combined use of PDT with immune stimulation treatments might reduce this action and improve the efficiency in defeating the local cancer and reducing cancer relapse as well as the onset of micro metastases, and increase the global effectiveness of anti-tumour treatment.

A different possibility to optimise the effectiveness of the PDT is the option of changing the structure of the PS. Rose Bengal (RB) is a xanthine dye-based PS with a configuration analogous to that of the biological probe fluorescein, but where all but two of the ring hydrogen atoms have been replaced by the halogen atoms chlorine and iodine. The second one offers a significant atom effect, which, due to increased spin-orbit coupling, stimulates intersystem crossing in the excited PS, causing an increased triplet state population and, therefore, ROS production.

Dhillon et al. covalently connected RB to the amphipathic peptide (AMP) C(KLAKLAK)2 and evaluated the efficacy of the resultant RB-C(KLAKLAK)2 conjugate as a PDT sensitiser. The RBC(KLAKLAK)2-mediated PDT of subcutaneous B16-F10-Luc2 cancer in C57 mice caused a greater reduction in cancer lesions with respect to controls. The synergistic action between RB and C(KLAKLAK)2 has been ascribed to the AMP sensitising cells to ROS, making them more vulnerable to ROS-provoked oxidative stress [132].

An additional optimisation of the therapeutic effects of PDT could be achieved via the contemporaneous use of different elements capable of stimulating oxidative stress. Remarkable data can be obtained from the studies performed using fullerenes. Fullerenes are the third major carbon allotropes after diamond and graphite [133]. They solely consist of n three-coordinate carbon atoms that are organised in precisely 12 pentagons and (n/2- 10) hexagons [133]. Fullerenes have several specific characteristics, such as being lightweight, chemical stability and conductivity. The most studied form of fullerene is fullerene C60 (C60). C60 is made of 60 carbon atoms establishing a symmetrical truncated icosahedron. C60 is an extraordinarily stable molecule of ~0.7 nanometres, with a molecular mass of 720 g/mol. C60 has a unique dual property. In fact, it can operate as a radical scavenger or as an oxygen radical producer. It is recognised to work as a radical sponge or antioxidant because of its 30 carbon double bonds to which multiple radicals, including ROS, can be attached [134]. Notably, fullerenes do not turn out to be a self-reactive free radicals during this event unlike most other antioxidants, including ascorbic acid and α- tocopherol. For this reason, C60 is one of the strongest radical scavengers [135]. However, in the presence of molecular oxygen, C60 is capable of producing ROS such as the superoxide anion through a type I (electron transfer) reaction and singlet oxygen through a type II (energy transfer) pathway. Nevertheless, the original C60 is biologically inactive, as it is hydrophobic, and it is practically insoluble in polar solvents. Procedures to overwhelm this condition are the adjunction of hydrophilic functional groups to the C60 cage or the combination of C60 with water-soluble elements.

Several C60 by-products are employed in skin care products, but they have not been introduced in clinical dermatology until now. However, due to its specific characteristics, in the future, C60 could be used as an agent in conditions where oxidative stress could have a therapeutic action such as PDT [136].

Finally, topical therapy employing PDT for several types of skin cancer has mainly been limited by the inability of PSs to pass through into the profound skin tissue. One option is the use of a different method of PS release. For instance, a new technique of PS delivery, which augments cancer tissue selectivity, is the utilisation of molecular carriers, such as liposomes, nanoparticles and ethosomes [137,138]. Several cationic liposome-based preparations having chlorine-based Foscan revealed elevated selectivity for cancers. It is important that these carriers improve general tissue diffusion, mainly as far as hydrophilic PSs such as ALA [137].

Tham et al. employed a mesoporous nanovehicle with the dual loading of PSs and drugs for combination treatment, while employing microneedle technology to improve their penetration into deep skin tissue [139]. Sub-50 nm photodynamically active mesoporous organosilica nanoparticles were produced with PSs covalently linked to the silica matrix, which enhanced the quantum yield and photostability of these PSs. The mesopores of the nanoparticles were also charged with small-molecule inhibitors (trametinib and dabrafenib) able to affect the MAPK pathway for melanoma therapy. These preparations demonstrated a synergistic killing action on skin tumour cells mainly via ROS and caspase-activated apoptosis [139].

Up-conversion nanoparticle (UCNP)-based photosensitisers have been presented as an emerging technology, which can overcome several limitations of conventional photosensitisers. When irradiated by low-energy NIR light, 31–45 UCNPs can emit high-energy photons (UV-vis and NIR). The use of NIR light as an irradiation source has its inherent advantages, including the key adjective minimisation of photodamage to biological tissue; an exceptional signal-to noise ratio, together with enhanced detection sensitivity owing to the inexistence of autofluorescence; and the ability to combine long wavelengths with conventional PSs [140].

A different approach has been attempted by Pucelik et al. [141]. The therapeutic efficacy of PDT with redaporfin (a fluorinated sulfonamide bacteriochlorin, F2BMet or LUZ11) was enhanced employing Pluronic-based (P123, F127) preparations. In vitro investigations against B16F10 melanoma cells demonstrated that redaporfin-P123 micelles increased oxidative stress with respect to redaporfin-F127 or PS alone. ROS-sensitive fluorescent probes demonstrated that the enhanced oxidative stress is owed, at least in part, to a more effectual generation of hydroxyl radicals, with an increase in the light-dose dependent cell death due to apoptosis or necrosis. Redaporfin in P123 was most efficacious in the PDT of C57BL/6J animals carrying subcutaneously implanted B16F10 melanoma cancers with no cancer relapse for over one-year post-therapy. These results showed that the preparation of redaporfin with Pluronic block copolymers overwhelms the resistance of melanoma cells to PDT increasing ROS production.

Recently, efforts to use semiconductor quantum dots (QD) as possible novel PSs have been made. In fact, ideally, they might be able to produce singlet oxygen directly via TET (triplet energy transfer) or indirectly via FRET (Förster resonance energy transfer) by stimulating PS molecules linked with them [142,143]. This approach could guarantee better results in the cytotoxic activity of PDT.

In the future, the know-hows applied for PDT could be also employed in diagnostics and the prognostic stratification of neoplastic diseases. For instance, cathepsins are a group of the cysteine protease family. Cathepsins control several biological responses including epidermal homeostasis, immune system responses, inflammation and angiogenesis. Three cathepsins, B, L and S, were demonstrated to have increased levels in several cancers. Moreover, cathepsins B and L were also discovered to be correlated with a worse prognosis and shorter survival of cancer subjects. Cathepsins were demonstrated to be extremely present and effective in tumour-associated macrophages (TAMs), which have an essential role in the progression of cancers [144].

Recently, experimental models were implemented with small molecule quenched activity-based probes (qABPs) that fluoresce upon activity-dependent covalent modification, yielding cell killing by PDT. The most efficacious and durable PS-qABP, YBN14, is based on a specific cathepsin recognition sequence, a QC-1 quencher and a novel bacteriochlorin derivative as a PS. YBN14 allowed the prompt and non-invasive in vivo imaging of subcutaneous cancers and provoked TAM death after light exposure. These data revealed that the PS-qABPs technique might be a useful diagnostic tool and could perhaps be employed in the PDT treatment of skin cancers [145]. Moreover, this approach could significantly decrease PSs’ light damage to skin tissue since the free unbound probe is still linked to the quencher that absorbs the energy, avoiding off-target ROS production [146].

One more emerging area of PDTs worth investigating is combination therapies, where a PS can be employed as a multifunctional drug or in combination with other anticancer and immune-suppressive drugs, to provoke synergistic or additive actions, and overwhelm cancer resistance.

However, the fields to be explored remain wide, and countless properties of UV exposure must be explored—for instance, the complex functions coordinated by the cutaneous neuro-endocrine system. Such effects are secondary to the transduction of UV electromagnetic energy into chemical, hormonal and neural signals. The locally induced cytokines, corticotropin-releasing hormone, urocortins, proopiomelanocortin-peptides, enkephalins or others can be released into circulation to exert systemic effects, including the activation of the central hypothalamic–pituitary–adrenal axis, opioidogenic effects and immunosuppression. Thus, UV touches the brain and central neuroendocrine system to reset body homeostasis [147].

Another aspect worth exploring is the relationship between exposure to ultraviolet rays and Vitamin D. In fact, for most people, more than 90% of their Vitamin D is produced endogenously from the exposure of the skin to solar UVB light (280–320 nm), synthesising the cutaneous production of precursors to 25-hydroxyvitamin D [25(OH)D]. It has indeed been shown that the pretreatment of keratinocytes with 1,25(OH)_2_D_3_ or CYP11A1-derived Vitamin D_3_ protected them against UVB-induced damage via the activation of the Nrf2-dependent antioxidant response and p53 phosphorylation, as well as by the induction of the DNA repair system [148].

Finally, we will have to investigate the role played by melanocytes and melatonin in photoprotection. Melatonin is the main neuroendocrine secretory product of the pineal gland. Although melatonin is best known to control circadian rhythmicity and skin pigmentation, the full spectrum of the functional properties of this free radical-scavenging substance includes the stimulation of complex antioxidative and DNA repair systems, and several immunomodulatory, thermoregulatory and anti-tumour properties. Moreover, melatonin protects human epidermal keratinocytes against UVB, and this property makes melatonin an especially attractive candidate agent in the future management of photo-induced damage [149].

In conclusion, with all the dares forward, it is our conviction that standardisation in future PDT investigation is of great relevance and is perhaps even more essential than the detection of novel PSs. In fact, just the accessibility to several and different PSs and diverse light sources makes PDT an intricate and difficult therapeutic procedure, which must be assigned to expert clinicians since in the majority of subjects, it needs to be adapted to every specific patient to adequately increase the overall efficacy of the PDT as an anti-skin cancer therapy.

## Figures and Tables

**Table 1:** Lesions and biological responses associated with Ultraviolet (UV) radiation and photodynamic therapy (PDT) (in ***bold italics*** are the mechanisms enabling tumour challenging; in *italics*, those having both pro- and anti-tumorigenic effects). Reactive oxygen species, ROS; extracellular matrix, ECM; malondialdehyde, MDA; thiobarbituric reactive substances, TBARS; 5,10,15,20-tetrakis(4-methoxyphenyl)-porphyrin, TMPP.

**Lesions**	
**Skin erythema**	
Vasodilation	
Inflammation	
Immunosuppression	
Dermatoheliosis	
Epidermal hyperplasia	
Skin carcinogenesis	
**Biological Response**	
**Response**	**Mechanism(s)**
ROS production	UV radiation causes extreme quantities of ROS that overcome antioxidant systems; ECM proteins act as photosensitisers producing ROS after UV irradiation
Tumorigenesis	ROS promote altered cell growth, DNA damage and epigenetic modifications, and cause the onset of tumours
***Cell proliferation block***	***MAPK and PI3K pathway activation***
***Cell death***	***MAPK and PI3K pathway activation***
***Necrosis***	***Cytosolic constituents spill into the extracellular space through the damaged plasma membrane and provoke a robust inflammatory response, in turn potentiating immunity by attracting host leukocytes into the tumour and increasing antigen presentation***
***Apoptosis***	***Minor oxidative stress induces apoptosis***
*Autophagy*	*Selective destruction of cellular elements* via *ROS*
*Innate immune system stimulation*	*Higher concentration of TNF-α and MDA, related to histological modifications due to ROS production*
*Lipid peroxidation*	*Modification of the ratio in the stimulation of cell death by apoptosis or by necrosis.* 5,10,15,20-tetrakis(4-methoxyphenyl)-porphyrin (*TMPP PDT*) *and zinc complex TMPP PDT provoked an augmented concentration of* thiobarbituric reactive substances *in tumour tissue and in blood plasma at 24 h after the PDT*
